# Relative permeability for water and gas through fractures in cement

**DOI:** 10.1371/journal.pone.0210741

**Published:** 2019-01-23

**Authors:** Kenton A. Rod, Wooyong Um, Sean M. Colby, Mark L. Rockhold, Christopher E. Strickland, Sangsoo Han, Andrew P. Kuprat

**Affiliations:** 1 Energy and Environment Directorate, Pacific Northwest National Laboratory, Richland, Washington, United States of America; 2 Division of Advanced Nuclear Engineering/ School of Environmental Engineering, Pohang University of Science and Technology (POSTECH), Pohang, South Korea; 3 Earth and Biological Sciences Directorate, Pacific Northwest National Laboratory, Richland, Washington, United States of America; Huazhong University of Science and Technology, CHINA

## Abstract

Relative permeability is an important attribute influencing subsurface multiphase flow. Characterization of relative permeability is necessary to support activities such as carbon sequestration, geothermal energy production, and oil and gas exploration. Previous research efforts have largely neglected the relative permeability of wellbore cement used to seal well bores where risks of leak are significant. Therefore this study was performed to evaluate fracturing on permeability and relative permeability of wellbore cement. Studies of relative permeability of water and air were conducted using ordinary Portland cement paste cylinders having fracture networks that exhibited a range of permeability values. The measured relative permeability was compared with three models, 1) Corey-curve, often used for modeling relative permeability in porous media, 2) X-curve, commonly used to represent relative permeability of fractures, and 3) Burdine model based on fitting the Brooks-Corey function to fracture saturation-pressure data inferred from x-ray computed tomography (XCT) derived aperture distribution results. Experimentally-determined aqueous relative permeability was best described by the Burdine model. Though water phase tended to follow the Corey-curve for the simple fracture system while air relative permeability was best described by the X-curve.

## Introduction

Relative permeability measurements are critical to understanding multiphase flow (e.g. gas and water) in subsurface environments where well drilling for operations such as deep carbon dioxide storage or geothermal energy production are employed [1–3]. Typically research on relative permeability has focused on natural sediment or rock, including fractured rock where fracturing occurs from subsurface geomechanical processes. However, cement used in the wellbore annulus is also subject to the same environmental stresses that result in fracturing of rock. Therefore, consideration of the effects of fracturing on the permeability and relative permeability of wellbore cement is also needed.

Cement is commonly used in the annulus of wellbores to seal where wells have penetrated, to prevent unwanted leaks from reservoirs into aquifers. Few geologic carbon storage studies to date have evaluated wellbore cement integrity in the field, but those which have noted that the use of Portland cement is effective for sealing aquifers against leaks due to its low permeability [4, 5]. Portland cement mixtures can be subject to enhanced physical and chemical degradation when used for CO_2_ storage and geothermal exploitation [4–10]. Eventually the extreme conditions of subsurface environments may impair cement mechanical integrity, leading to the propagation of fracture networks, and increasing the permeability for gas and water [11, 12]. To improve predictions of well leaks through fractured wellbore cement accurate flow models are necessary.

Since relative permeability can vary dependent on the solid state conditions it is significant that most work to date has focused on relative permeability through granular porous media, compared to the less investigated fractured media. Investigations of relative permeability of fractures have been conducted using natural rock, but they also often employ synthetic conditions such as the use of glass plates or clay bricks [13–16]. The variability of these measurements have been summarized in detail by Huo and Benson (16), and demonstrated that relative permeability models fit to data can depend on surface roughness of the material and the geometry of the fracture. To date, to the authors’ knowledge, there have been no reproducible experimental measurements of relative permeability for air and water through fractured wellbore cement published. The objective of this research is to determine the relative permeability for air and water through fractured cement for two different sets of fractures in class H Portland cement paste (cement) cylinders and to assess the appropriateness of different models (e.g. X-curve, Corey-curve, and Burdine) commonly used to describe relative permeability.

## Methods and materials

### Materials/Fracturing

**Fig 1:**
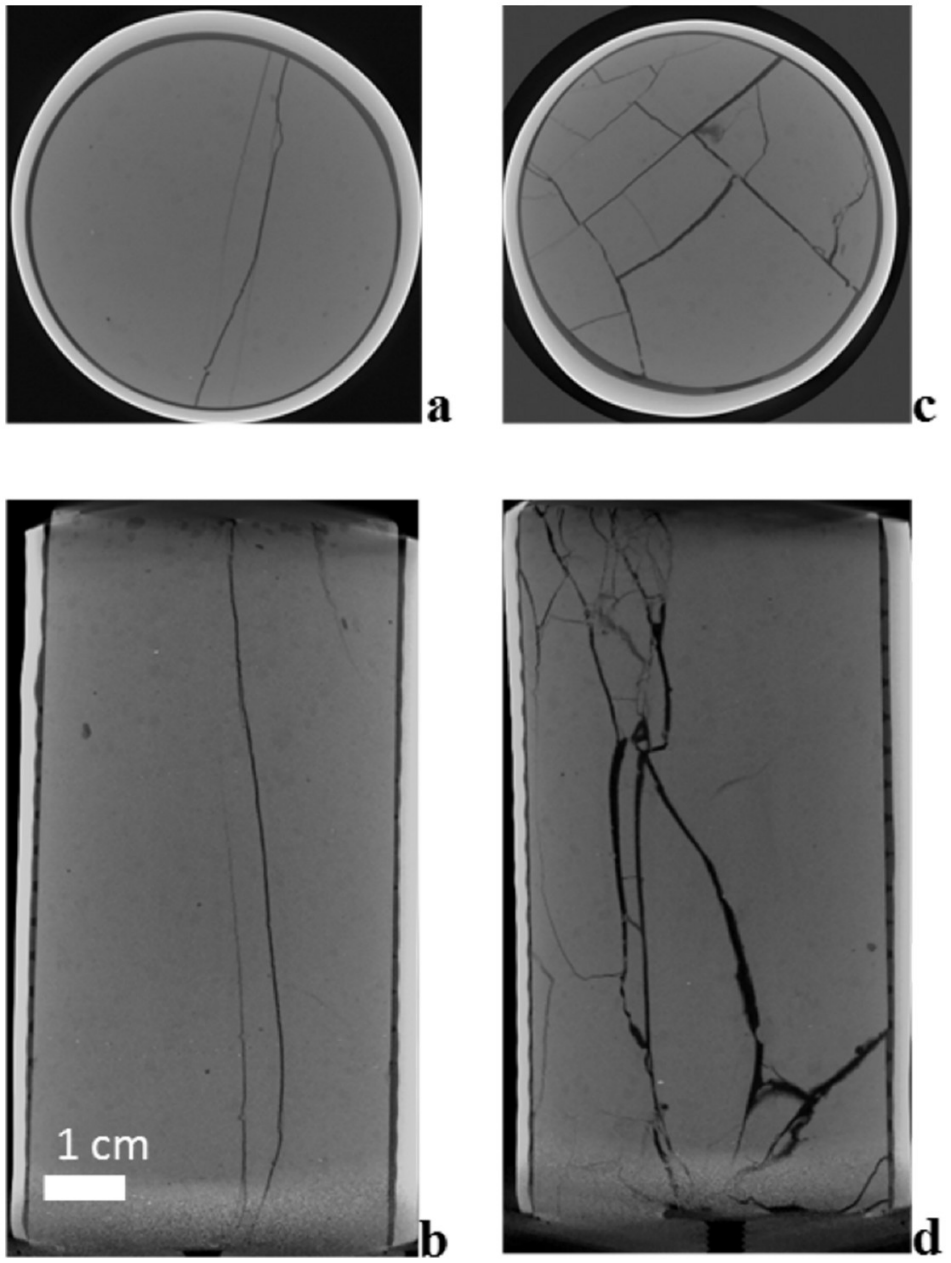
XCT cross section (a, c) and profile (b, d) of cement cylinders with simple fracture (a, b) and with multiple fractures (c, d). For both cylinders: diameter = 5.08 cm; length ≈ 10 cm. Medium grey is cement (median intensity 120), black lines in the cement are fractures (intensity between 60 and 100), black/dark ring around the edges of the cylinder are polymer/glue (median intensity 88), and light grey ring is heat shrink tube (median intensity182). The background has median intensity 22.

Cement paste cylinders with a length of 9.75 cm and diameter of 5.08 cm were fabricated. Cement samples used ordinary Portland cement (OPC; class H) with a water to cement ratio of 0.4 and blended for 15 minutes at 300–500 RPM with an overhead mixer, which had 5 cm diameter cross blade impeller. The slurry was poured into a plastic mold, vibrated to remove any air bubbles, and cured at 100% relative humidity for 28 days at 22°C. After removing the cured cement samples from the molds, the sides of cured samples were bound with heavy duty moisture-seal heat-shrink tubing, leaving the ends open (Fig 1). The tubing was used, shrunk to the cylinder sides, to hold the sample together after being fractured.

These cement paste cylinders were then placed in a hydraulic press with the flat ends half covered with offset metal plates such that a shear force could be applied down the length of the sample. Force (444 N and 667 N) was applied to both of the cylinders with the offset plates until a shear fracture occurred. This simulates a shear fracture which could occur with incomplete cementing of a wellbore combined with high pressures [17]. For the multiple fractures the cement sample was rotated 90° and fractured again, to increase the aperture and the number of fractures. The total volume of the fracture network in each cylinder was determined gravimetrically by weighing before and after imbibing water into the fractures for 24 hours. Samples were then allowed to saturate for one week before being drained and measured again for imbibing water. The calculation assumes no measured water imbibition into the cement matrix.

### X-ray computed tomography (XCT) and fracture segmentation

To characterize the internal fractures, the cement paste cylinder samples were scanned using a high-resolution microfocus XCT scanner (X-Tek/Metris XTH 320/225 kV). Scans were performed at 98 kV and 536 μA X-ray energy ranges with a 0.1 mm Cu filter. During the scans samples were rotated continuously with momentary stopping to collect each projection. A total of 1,998 projections were collected over 360 degrees with an isotropic voxel resolution of 58.4 μm, resulting in 8-bit grey-scale XCT images (Fig 1).

The code used to perform the automated fracture segmentation was written in Python 2.7 and relies upon OpenCV [18] and NumPy [19]. The approach follows that of Tang [20], but with application-specific modifications.

Images were initially processed by a simple moving median filter with window size *m* = 10, wherein images in the range *[i–m*, *i + m]* are used to calculate the median for each pixel in image ***i***. This process assumes features (i.e., the fracture) change insignificantly within the window such that features are preserved and high-frequency elements (noise and artifacts) are ameliorated. When applied, signal-to-noise ratio (SNR) is improved, and certain imaging artifacts (e.g., ring artifact) are removed. A NumPy implementation of anisotropic diffusion was then applied to each image *i* to further increase SNR [21].

An initial segmentation of the fracture was performed using adaptive thresholding [22]. Local thresholds were calculated using a Gaussian-weighted kernel 101 pixels wide. Pixels of intensity 3 lower than their respective local threshold were selected, effectively resolving the relatively darker areas of each image (Fig 1). Morphological operations were then applied successively to fill small gaps in the segmentation and to remove noise.

To isolate the fracture and exclude the empty space around the sample, a coarse region of interest (ROI) extraction was performed. Images were thresholded by pixel intensity, approximately, between grey-scale values of 100–130. Morphological operations were again applied to remove noise and to fill any areas missed within the ROI. The initial segmentation was multiplied by this coarse mask to exclude any areas outside the mask. Finally, small components were removed using connected-components analysis [23].

A variant of the marching cubes algorithm [24] was then used to extract a triangulated surface mesh from the segmented data. Volume-conserving smoothing [25] was employed to smooth surface normal. Fracture dimensions and other properties were determined using the Los Alamos Grid Toolbox [26].

### Electrical resistivity measurement

Fracture water saturation was monitored in the cement cylinders by electric resistivity measurement using a two electrode method [27–29]. The fractured cement samples were fitted with stainless steel end caps using heavy duty moisture-seal heat-shrink tubing over the original heat shrink wrap. The seal was further reinforced using ring clamps. Electrodes were connected to stainless steel pressure fittings in the end caps, such that current could pass through the cement.

An initial calibration was conducted before relative permeability tests (S1 Table, S1 Fig; supporting information), and the slope of the resistivity versus ratio fracture saturation (gravimetric) curve, was used to determine effective saturation of the fracture. The calibration was done using the same solution (0.1M NaNO_3_, pH = 7) used in the experiments. The cement samples were maintained wet at all times so that weight changes due to absorption of water into the cement matrix during the calibration and test would be below detection limits. Because of this the changes in the moisture content measured corresponded to fracture saturation.

Before conducting each relative permeability test the electrical resistivity of water-saturated fracture and water-unsaturated fracture was checked to ensure there was no drift in the calibration. For checking impedance at full water saturation of the fracture, measurements were made while water was flowing from bottom to top until all air effectively was eluted from flow in the vertically oriented cylinder. For checking impedance of the water-unsaturated condition measurements were taken with air only being supplied to the cylinder and flowing from top to bottom until the water had effectively drained from the fracture. In both cases values were recorded once the voltage response stabilized.

### Permeability measurement

Absolute permeability of fractures was determined before relative permeability tests were performed. Flow was supplied to the influent end through 0.125 inch (3.175 mm) polytetrafluoroethylene (PTFE) tubing attached with two pressure fittings, in ports of the stainless steel end cap, one for water and the other for air flow (S2 Fig). The end cap was etched with flow channels on the surface in contact with the cement to ensure water and/or air would disperse over the surface and enter the fractures evenly.

Fracture absolute permeability (solution saturated) was measured using saturated constant head method [30]. Constant pressure to the influent end was controlled by maintaining a solution reservoir (sealed vessel) at a constant pressure, between 111–141 kPa, and discharge was measured at atmospheric pressure. Pressure was maintained constant using precision differential air flow controllers (PCD series controller, Alicat Scientific, Tucson AZ).

The Reynolds equation (Eq 1) describes flow through fractured media [31] and can be used with measured data from experimentation to calculate the effective fracture aperture (*b*) dimension. When combining the Reynolds equation (Eq 1) with Darcy’s equation (Eq 2), the equation reduces to a form which can be used to calculate the equivalent permeability based on fracture aperture (Eq 3):

$$Q=-\frac{Wb^{3}}{12\mu }\left(\frac{h_{i}-h_{o}}{L}\right)$$ (1)

$$Q=-\frac{k_{e}Wb}{\mu }\left(\frac{{hP}_{i}-{Ph}_{o}}{L}\right)$$ (2)

$$k_{e}=\frac{b^{2}}{12}$$ (3)

where *Q* = discharge (cm^3^ s^-1^); *W* = effective fracture width (cm); *b* = effective aperture of fracture (cm); *μ* = viscosity (Pa·s); *h*_*i*_ = pressure at inlet (Pa); *h*_*0*_ = pressure at outlet (Pa); *L* = length of fracture (cm); *k*_*e*_ = effective permeability (cm^2^).

For the calculations the effective fracture width (*W*) used was the average width (5.21 mm) determined for cement cylinder with the simple fracture by measuring the fracture, which ranged from 5.08 mm to 5.33 mm. Since the cement cylinder with multiple fractures had a complex fracture network, a representative fracture width was not possible to determine, and as such the width of the cylinder (5.08 mm) was used for the effective fracture width. This was determined to be a preferred compromise since there was at least one location in the cylinder where the fractures coalesce and the width of the cylinder is a close representation of the fracture width.

### Relative permeability measurements

Relative permeability experiments were conducted by introducing both aqueous solution (0.1M NaNO_3_, pH = 7) and air to the influent end of the cylinder simultaneously at a constant pressure with the cylinder oriented horizontally or vertically with flow from top to bottom. Pressure applied for gas and aqueous phase was averaged to calculate influent pressure, with the upstream pressure stepped up or down within the range 108–120 KPa. Measurements were made when the flow was at steady state and the pressure constant. The effluent PTFE tubing emptied into a stoppered flask used as a phase separator. This flask was positioned on a precision scale where change in effluent mass was recorded over time. Effluent air flow was measured using a precision air flow meter (M series meter, Alicat Scientific, Tucson AZ). Air and 0.1M NaNO_3_ solution were selected for performing the tests to minimize reactions with the cement paste, which could change the fracture aperture through precipitation or dissolution reactions [32].

Relative permeability through fractures was calculated using the determined permeability (*k*_*e*_) and Eqs 4 and 5 [33]. Eq 4 was used to calculate aqueous permeability, *k*_*rw*_, and Eq 5 was used for air permeability, *k*_*rg*_, to compensate for compressibility of gas.

$$Q_{w}=\frac{k_{e}k_{rw}A(h_{i}-h_{o})}{\mu_{w}L}$$ (4)

$$Q_{g}=\frac{k_{e}k_{rg}A(h_{i}^{2}-h_{o}^{2})}{\mu_{g}LP_{o}}$$ (5)

where *Q*_*w*_ = water discharge (cm^3^ s^-1^); *k*_*rw*_ = relative permeability of water (dimensionless); *A* = cross section area of fracture aperture and width (cm^2^); *μ*_*w*_ = dynamic viscosity of water (Pa·s); *Q*_*g*_ = gas discharge (cm^3^ s^-1^); *k*_*rg*_ = relative permeability of gas (dimensionless); *μ*_*g*_ = dynamic viscosity of gas (Pa·s). Raw data can be found in the supporting information.

An additional test was performed to reduce the gas phase interference during multiphase flow. For this test, solution was applied in the same way as above but the fracture was oriented vertically. Solution saturation was controlled by allowing gravity drainage, reducing the water flow rate, and maintaining sufficient air pressure to drain the fracture but having only the aqueous phase flow out of the fracture.

Irreducible water content was determined by adjusting the pressure for air and water to the point where only air was flowing through the fracture then recording the mass and resistivity information for this point. The irreducible water content would be comprised of the surface moisture in the main fracture channel and water in smaller less hydraulically connected channels.

### Relative permeability models

Fluid flow in porous and fractured media can be modeled using many different approaches, and permeability and relative permeability can be calculated from computed fluxes and pressure gradients using Darcy’s Law. Blunt, Bijeljic (34) reviewed methods for pore-scale imaging and modeling and noted that the most popular method for computing single and multiphase flow directly on pore-space images is the lattice Boltzmann method [35–40]. Pore network models are also very popular owing to their relative simplicity and computational efficiency [34]. Also popular, but less well-used owing to increased computational demand, is directly solving the Navier-Stokes equation on discretized representations of the pore space [41]. Techniques referred to as density functional modeling, including smooth particle hydrodynamics, have also been used for modeling fluid flow in porous media [42, 43]. All of these methods can use information about the pore space obtained from XCT images [35], or thin sections [44], to compute fluid fluxes under specified pressure gradients and to estimate permeability and relative permeability. However, when flow models are being incorporated into larger risk assessment models for well operations, then relatively simple models describing fluid flow would be preferred for the single component of wellbore relative permeability.

Models used for comparison with data include the X-curve and Corey-curve [15, 45]:

X-curve:

$$k_{rw}=S_{e}$$ (6)

$$k_{rg}=({1-S}_{e})$$ (7)

To adjust Corey-curve for irreducible water (the lowest achievable water saturation from displacement by the gas phase):

$$S_{e}=\frac{\left(S-S_{wr}\right)}{\left(1-S_{wr}\right)}$$ (8a)

To adjust Corey-curve for irreducible water and irreducible gas:

$$S_{e}^{*}=\frac{\left(S-S_{wr}\right)}{\left(1-S_{wr}\right)}+S_{gr}$$ (8b)

Where *S*_*e*_ = effective water saturation; S*_e_ = apparent water saturation; *S* = water saturation; *S*_*wr*_ = residual water saturation; *S*_*gr*_ = residual gas saturation

Corey-curve:

$$k_{rw}=S_{e}^{4}$$ (9)

$$k_{rg}={\left(1-S_{e}\right)}^{2}∙(1-{S_{e}}^{2})$$ (10)

The X- and Corey-curves are appealing due to their simple forms, but they may or may not provide accurate estimates of relative permeability. Aside from porosity, which is needed for calculating saturation, the X-curve requires no additional parameters while the Corey-curve requires a single parameter, the residual wetting fluid saturation, *S*_*wr*_. Brooks and Corey (46) note that the approximation used by Corey is only valid for a particular pore-size distribution. Therefore more accurate relative permeability estimates might be obtained if consideration is given to the pore, or for fractured media, the aperture size distribution.

Wang and Narasimhan [47] developed a statistical theory for fluid flow in partially saturated, rough-wall, variable aperture fractures in which the cube of the single value of aperture in Eq 1 is replaced by

$${\langle b^{3}\rangle }_{1}=\int_{0}^{b_{max}}b^{3}f(b)db$$ (11)

where *f*(*b*) is the aperture size distribution and the subscript 1 is used to denote fully saturated conditions. For partially saturated (air-water) conditions,

$${\langle b^{3}\rangle }_{S}=\int_{0}^{b_{S}}b^{3}f(b)db$$ (12)

where

$$b_{S}=-\frac{2\gamma cos\theta }{\rho gh}$$ (13)

and where γ is the interfacial tension between non-wetting and wetting fluids (air-water), θ is the contact angle between the solid and wetting fluid, and h is the capillary pressure (in units of water-equivalent hydraulic head). Values of interfacial tension, aqueous density, and contact angle for an air-water system of 0.07183 kg s^-2^, 1000 kg m^-3^, and zero degrees were assumed. Wang and Narasimhan [47] assumed that fracture aperture distributions could be represented by a one-parameter gamma function, based on results from Tsang [48] for natural fractures in granite, so that

$$f(b)=\beta^{2}(b+b_{c})e^{-\beta (b+b_{c})}$$ (14)

where *β* is a shape parameter and *b*_*c*_ is a cutoff aperture. The relative permeability, *k*_*r*_, and absolute saturation, *S*, of the fracture were then defined as

$$k_{rw}(h)=\tau \frac{{\langle b^{3}\rangle }_{S}}{{\langle b^{3}\rangle }_{1}}$$ (15)

$$S(h)=\frac{{\langle b\rangle }_{S}}{{\langle b\rangle }_{1}}$$ (16)

where τ was referred to as a phase-separation constriction factor that is computed numerically based on the fraction of area occupied by liquid water and fracture surfaces that are in contact [33; Eqs 7–10]. Aperture distribution parameters representing a fractured tuff were estimated by Wang and Narasimhan [47] using borehole-observable quantities, including apparent fracture frequency and dip angles, the fraction of contact area (inferred from the fraction of fracture surface area coated by zeolites, clay, and calcite), and the bulk permeability of the fracture continuum.

In the current study, the permeability of the fracture continuum for the concrete cylinders was determined experimentally, and the aperture distribution for the simple fracture was determined from image analysis of the segmented XCT data. Given an aperture size distribution, and the bulk permeability of the fracture continuum, the model of Wang and Narasimhan [47] can be used to generate discrete values of *k*_*r*_*(h)* and *S(h)*. The discrete values may be fit with more general models for constitutive relative permeability-saturation-capillary pressure relations in porous media [46, 49].

Brooks and Corey [46] showed that for a large number of experimental data sets the effective saturation of the wetting fluid was well represented by the equation

$$S_{e}={\left(\frac{h_{b}}{h}\right)}^{\lambda }for\;h\geq h_{b}$$ (17)

where *λ* characterizes the pore-size distribution, and *h*_*b*_ is the bubbling pressure, a measure of the maximum pore size forming a continuous network of flow channels within the porous medium. Brooks and Corey [50] used this relationship with the Burdine [51] model to derive the following relationships for the relative permeability of wetting and non-wetting fluid phases

$$k_{rw}={\left(S_{e}\right)}^{\frac{2+3\lambda }{\lambda }}$$ (18)

$$k_{rg}={\left(1-S_{e}\right)}^{2}(1-{S_{e}}^{\frac{2+\lambda }{\lambda }})$$ (19)

Fracture aperture distribution results, inferred from image analysis of XCT measurements, were used in conjunction with the model of Wang and Narasimhan (47) to estimate pressure-saturation relations for the fractured concrete. The estimated pressure-saturation values were then fit with the Brooks and Corey (46) model to estimate a pore-size distribution parameter used in the Burdine (51) relative permeability model. Calculated permeabilities from laboratory experiments are compared to results generated by different models (e.g. Corey curve, X curve, combined Wang and Narisimhan/Brooks-Corey/Burdine models) in Section 3.

Data generated from relative permeability measurements were compared to models using linear regression [52]. The linear regressions were fit to observed (*y*-axis) to predicted (*x*-axis) with the intercept set to the origin.

## Results and discussion

### Fracture characterization

**Fig 2:**
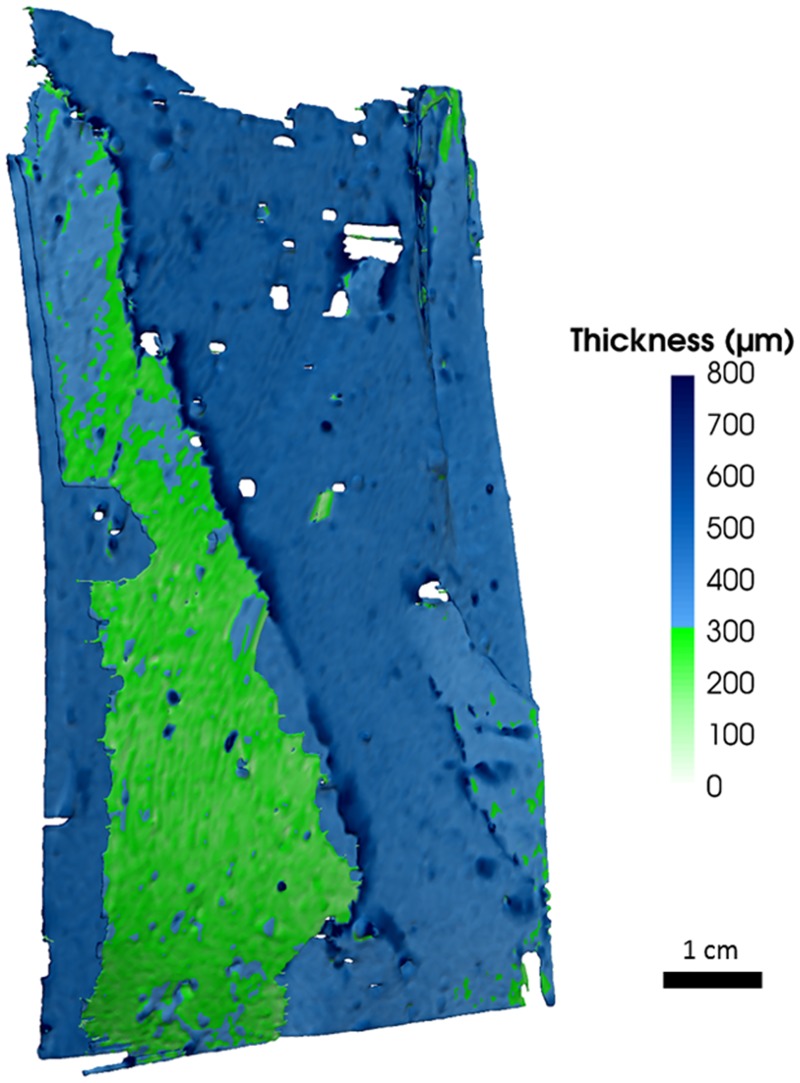
Computationally segmented primary continuous fracture from simple fracture cylinder. Color coding represents thickness of fracture aperture. Dimensions of the fracture are 5.08 cm x 9.75 cm. The restrictive aperture to flow is the smallest represented by green in the model. This green region includes apertures <300 μm, with most ranging 200–300 μm. Voxel size is 58.4 μm. Flow direction relative to the image was from top to bottom.

The simple fracture cylinder has one continuous major fracture across its width and length with only two smaller and partially connected branching fractures (Fig 1a and 1b). Within this matrix, the fully connected fracture had apertures of up to 0.8 mm, with 75% of the apertures ranging in size from 0.2–0.5 mm, and fracture volume of 2.4 mL based on computationally segmented fracture image analysis (Fig 2 and S3 Fig). This volume is lower than the total effective fracture volume of 3.9 mL based on gravimetric measurements, suggesting that approximately 1.5 mL of the fracture network are small channels that visually were evaluated to not be hydraulically significant and not evaluated by the segmentation. Across the fracture width there is a region (green and light blue) near the middle to bottom, where the narrowest apertures that are most restrictive to flow range in size from mostly 0.2–0.4 mm (Fig 2). This is more than double the effective aperture of 0.07 mm calculated using Eq 1 above, from water saturated permeability data experiments, assuming the fracture width is the same as the cylinder diameter. Due to a combination of smoothing of features during data processing and with a voxel size of 58.4 μm the surface roughness is not visible across the entire segmented fracture. In spite of this some texture can be seen in the image showing that the fracture is not smooth (Fig 2). The multiple fracture cylinder was not segmented due to the complexity of the fracture network.

S3 Fig shows the normalized, empirical cumulative aperture distribution determined for the simple fracture in the concrete cylinder, computed from XCT results, and a gamma function (Eq 14) fit to the XCT image-derived aperture distribution results. For natural rocks, Wang and Narasimhan (47) suggested that the *b*_*c*_ parameter be estimated from the fraction of fracture surfaces coated by clay minerals, zeolites, and calcite. This type of information is not applicable to the concrete used in this study so a value of 0.0 was assumed for *b*_*c*_. A value of 0.0026 μm was estimated for the *β* parameter by fitting the fracture aperture distribution data generated from analysis of the XCT data. S3 Fig indicates that the experimentally-derived aperture distribution for the simple fracture is well represented by a gamma function. According to the computed XCT results, the maximum aperture in Eq 11, *b*_*max*_, for the simple fracture is 0.8 mm. The mean aperture, estimated from the cube root of 〈b^3^〉_1_ in Eq 11, is 0.4347 mm. Using this mean aperture in Eq 11 yields a permeability value of 1.57×10^−4^ cm^2^, which is a factor of ~37 greater than the measured permeability of the sample for water saturated conditions. It is likely that a localized region of the fracture network where there is a narrowing of the aperture to less than 0.2 mm and where fluids must pass is producing the lower permeability.

In contrast to the simple fracture cylinder the multiple fracture cylinder has a network of intersecting fractures (Fig 1c and 1d). These fractures can be larger than 1 mm (Fig 1d) though most are of a similar magnitude to the simple fracture (Fig 1a and 1b). The multiple fracture cylinder effective pore volume was 9.9 mL, determined gravimetrically, which was over twice that of the simple fracture. Based on water discharge and assuming the same width of fracture as the simple fracture due to the same cylinder diameter, the calculated effective aperture is 0.26 mm for Eq 1, assuming a single fracture. However, because of multiple fractures providing a complex network which contributes to the permeability (Fig 1c), it can be seen that the fracture width and aperture have varying dimensions compared to the simple fracture.

### Relative permeability

#### Corey and X-curve

**Fig 3:**
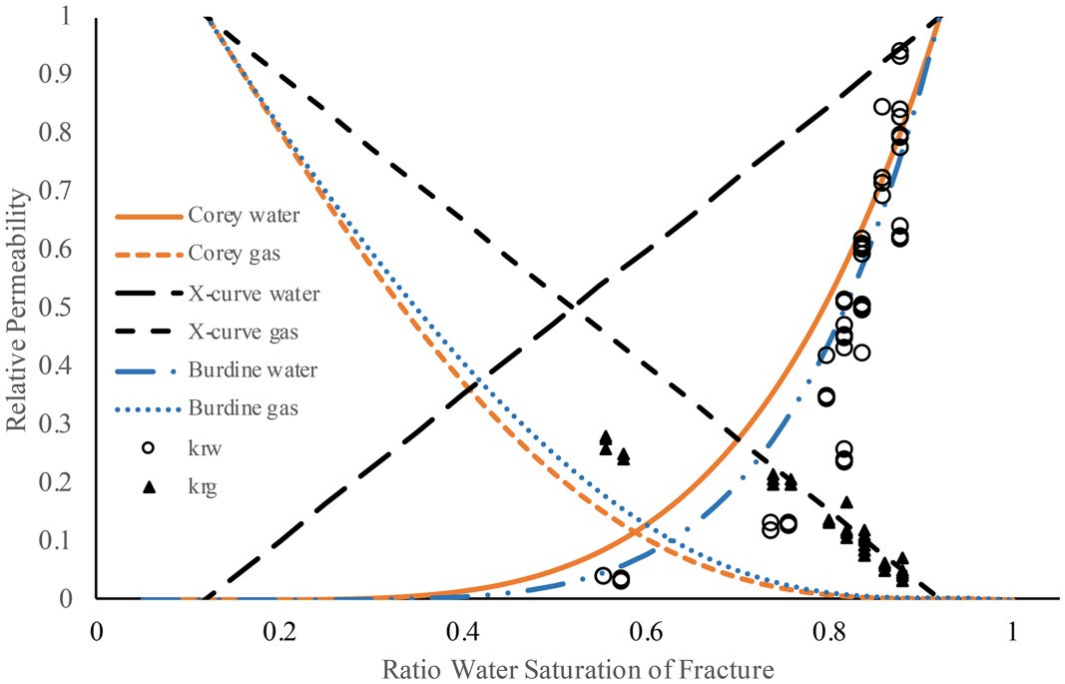
Relative permeability plot of simple fracture cement cylinder, models adjusted for both irreducible water and irreducible gas. Water (open circles; 0.1 M NaNO_3_; *k*_*rw*_) and gas (closed triangles; air; *k*_*rg*_) were from a paired relative permeability experiment with core oriented horizontally. Solid line and dashed orange line are the Corey-curve, dashed black lines are the X-curve, and blue dashed dot lines are the Brooks-Corey/Burdine curve respectively for relative permeability.

**Fig 4:**
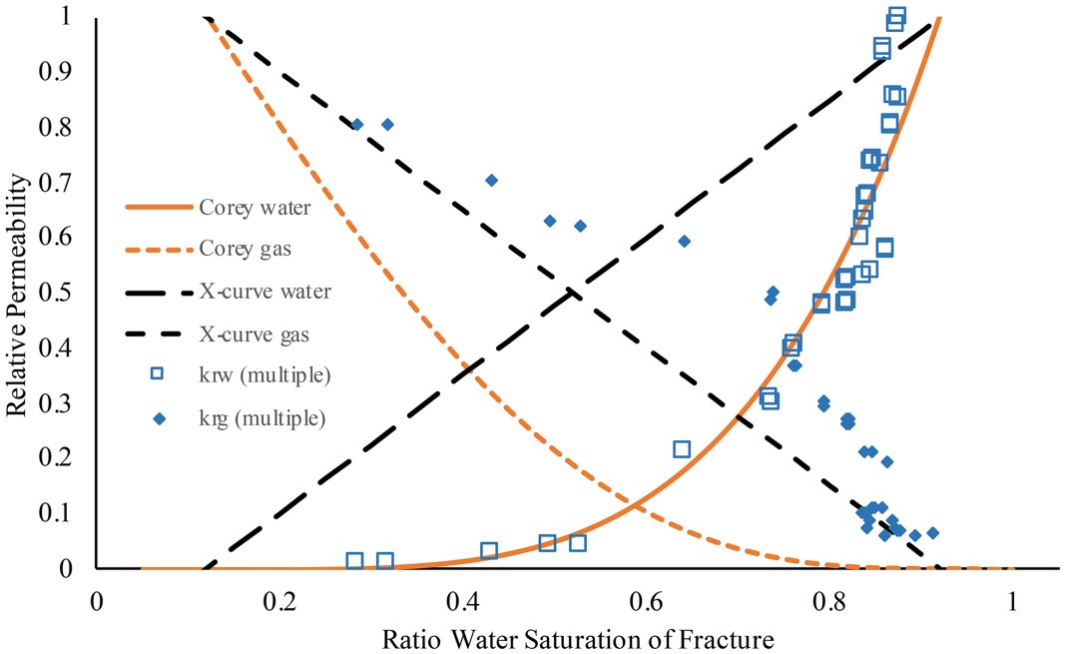
Relative permeability plot of multiple fracture cement cylinder. Water (open squares; 0.1 M NaNO_3_; *k*_*rw*_) and air (closed diamonds; air; *k*_*rg*_) were from a paired relative permeability experiment. Solid line and dashed orange line is the Corey-curve and dashed black lines are the X-curve respectively for relative permeability.

Relative permeability was tested on both simple fracture and multiple fracture cylinders (Figs 3 and 4). The measured relative permeability data was plotted as a ratio of the absolute permeability (simple fracture *k*_*e*_ = 4.19 × 10^−6^ cm^2^; multiple fracture *k*_*e*_ = 7.63 × 10^−6^ cm^2^) compared to Corey-curve and X-curve models. Since the irreducible water content was near 20% saturation, the curves were adjusted for this minimum saturation. Comparing between the cylinders, a similar trend was displayed for the relative permeability of water with a curve similar to that of a Corey-curve (Figs 3 and 4). Using linear regression of the measured data to the predicted Corey-curve the simple fracture had an r^2^ of 0.78 and the multiple fracture had an r^2^ of 0.90 (Table 1).

**Table 1:** Slope and coefficient of determination (r^2^) from linear regression comparing observed (data; *y*-axis)) to predicted (models; *x*-axis).

	Cylinder	
Model	Simple Fracture slope (r^2^)	Multiple Fracture slope (r^2^)
x-curve water	0.58 (0.35)	0.75 (0.57)
x-curve air	0.72 (0.74)	1.25 (0.84)
Corey curve water	0.85 (0.78)	1.03 (0.90)
Corey curve air	2.15 (-0.92)	1.76 (0.05)
Burdine curve water	0.94 (0.84)	n/a
Burdine curve air	1.82 (-0.86)	n/a

n/a = not applicable; perfect fit would be slope = 1.0 with r^2^ = 1.0

With both of the cylinders, the relative permeability of water reaches absolute permeability near 90% saturation rather than at 100% saturation (Fig 3). This makes the measured data points which form the curve near 90% water saturation appear to be shifted to lower saturation than expected, and this shift appears similar to what can be found when a non-wettable fluid is transported [1]. However, cement generally is water wettable [53] so it is not likely that wettability of water would cause this shift in pattern. More likely, at close to 90% effective water saturation the gas phase is having a minimal interference on flow of the water phase, leaving the Corey-curve model, without adjustments for trapped air, to become an underestimate of the permeability at this saturation. This shift could be attributed to the heterogeneities in the fracture aperture with water flowing in the larger channels (up to 0.8 mm aperture) unhindered by the gas phase.

Relative permeability for air was described relatively well by the X-curve for both fracture systems (Figs 3 and 4) where the model fits the data with an r^2^ of 0.74 and 0.84 for the simple fracture and multiple fracture respectively (Table 1). This contrasting pattern between Corey-curve for aqueous phase and X-curve for gas phase has been found in stratified sedimentary environments where flow is parallel to the layers of stratification, but the flow pattern will change to Corey-curve only when multiphase flow is perpendicular to stratification [54] and therefore more subjected to porous media flow. The large aperture sizes of the fracture network (Fig 1c and 1d) would allow air to flow without measurable phase interference while at the same time the air flow is interfering with water flow in regions of the fracture network with smaller aperture, resulting in water flow more similar to that in non-fractured porous media [2].

#### Burdine model

To investigate if the Corey model could be improved upon, the Burdine relative permeability models were calculated based on values determined from the XCT-based aperture distribution (Fig 3; Table 1). The Burdine [37] parameters for the computed fracture saturation-pressure values that were determined from the Wang and Narasimhan [47] model using Eq 16 are *h*_*b*_ = 1.831 cm and *λ* = 1.0. These values were obtained by fitting discrete pressure-saturation values generated using Eq 16 (with terms defined by Eqs 12–14) with the Brooks-Corey [32] model, Eq 17. Note that only the *λ* parameter appears in the Burdine [37] relative permeability functions, Eqs 18 and 19. Use of the measured aperture distribution with the Burdine model, observed to predicted r^2^ of 0.84, appears to provide nominal improvement in correspondence between modeled and experimentally-determined relative permeabilities for the simple fracture network compared to the Corey model, observed to predicted r^2^ of 0.78 (Table 1).

#### Vertical flow and reduced air pressure

**Fig 5:**
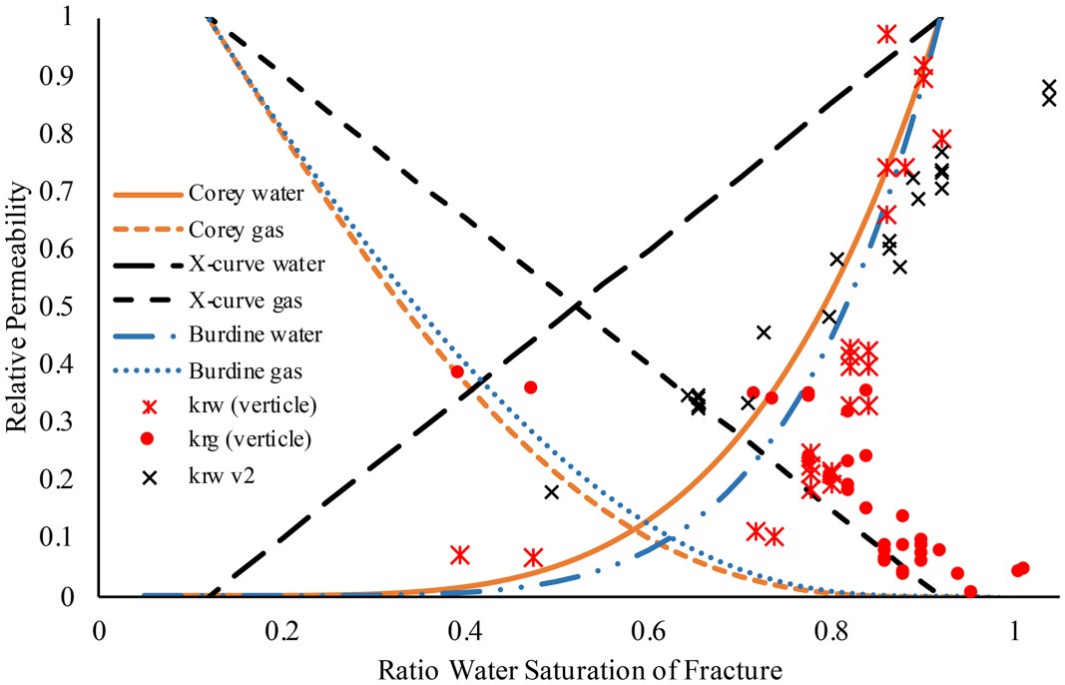
Relative permeability plot of simple fracture cement cylinder with fracture oriented vertically, models adjusted for irreducible water and trapped air. Water (asterisk; 0.1 M NaNO_3_; *k*_*rw*_) and air (closed circles; air; *k*_*rg*_) were from a paired relative permeability experiment. The second water plot (crosses; 0.1 M NaNO_3_; *k*_*rw*_ v2) was a second experiment where there was no effluent air phase, the only effluent was water Solid line and dashed orange line are the Corey-curve, dashed black lines are the X-curve, and blue dashed dot lines are the Brooks-Corey/Burdine curve respectively for relative permeability.

To investigate the influence of flow orientation and the interference of gas entrapment on water flow, unsaturated vertical water flow experiments were also conducted on the cylinders (Fig 5). These experiments were conducted by flowing water through the unsaturated fracture, with varying effective saturation (>40%), and with the cylinder oriented vertically to allow gravity flow, using minimal air pressure and adjusting water flow to assist gravity with desaturating the fracture. Settings were maintained to achieve steady state prior to measurements. By lowering the gas pressure and as a result reducing the phase interference of air entrapment on water flow, the relative permeability of water becomes more linear (*k*_*rw*_ v2 in Fig 5), and in the cylinder with multiple fractures the data more closely follows the X-curve, similar to a simple, uniform, smooth fracture with no phase interference [15]. However, using the same vertical orientation to measure relative permeability the result was similar trend to horizontal (Fig 5). This demonstrates how small adjustments to the air pressure can result in a dramatic change in measured relative permeability pattern.

## Conclusions

Experimental data on permeability and relative permeability of water and gas in fractured cement paste cylinders indicate that the most appropriate relative permeability model depends on the saturation state of the materials, and on the characteristics of the fracture network. In both simple and multiple fractured cement samples the Burdine model provided the closest correspondence to experimentally determined relative permeabilities, followed by the Corey-curve. The X-curve better described the relative permeability of the gas phase. If water pressure is maintained and gas pressure is reduced such that the phase interference is minimized, the relative permeability of water will more closely follow the X-curve. At greater than 80% water saturation, the interference of the gas phase on water permeability is reduced and water can easily permeate the fractures. The result is that a fracture when less than fully saturated, can have water relative permeability at maximum. This suggests that more water can be transmitted under these higher water saturation conditions than predicted by the models unless trapped air is accounted for in the calculations. Use of the aperture distribution for computing fracture saturation-pressure and relative permeability relations appears to yield some nominal improvement in correspondence between experimentally-determined and model generated relative permeability results for water.

## Supporting information

S1 Table Resistivity calibration data for fracture saturation of cylinders used. (DOCX)

S2 Table Raw data. Data measured from relative permeability experiments.
(DOCX)

S1 Fig Regression of resistivity saturation calibration from S1 Table. Blue triangles are simple fracture and black circles are multiple fracture sample.
(DOCX)

S2 Fig Schematic of experimental design. Monolith was wrapped in polyolefin tubing with stainless steel end caps (with influent and effluent ports) attached. Air pumps supplied air to precision flow controllers, where one supplied air directly to the monolith influent end cap and the other to a sealed reservoir. Head pressure to the influent end was controlled by maintaining the reservoir at a constant gauge pressure (10–40 kPa). Resistivity meter electrodes were attached to the steel pressure fittings on the end caps. Effluent eluted from one port into a beaker, which acted as a phase separator, on a scale. Beaker was sealed with a tube to an air flow monitor.
(DOCX)

S3 Fig Cumulative fraction of aperture size from computational segmented fracture and calculated gamma function, Eq 14. (DOCX)
