# Supplementary material for: Relative permeability for water and gas through fractures in cement
Source: PLoS One. 2019 Jan 23;14(1):e0210741. doi: 10.1371/journal.pone.0210741 (PMC6343898; doi:10.1371/journal.pone.0210741)
Supplement: S1 Table — (DOCX) [file pone.0210741.s001.docx]

**S1 Table**. Resistivity calibration data for fracture saturation of cylinders used.

| Sample | Frequency (KHz) | Angle (˚) | Impedance (Ω) | Mass sample  (g)* | Resistivity  (Ω·cm) | Ratio fracture saturation |
| --- | --- | --- | --- | --- | --- | --- |
| Simple fracture | 5 | 1 | 966.0 | 1693.4 | 1957.9 | 1.00 |
|  |  |  | 1050.0 | 1692.9 | 2128.2 | 0.88 |
|  |  |  | 1145.0 | 1691.1 | 2320.7 | 0.46 |
|  |  |  | 1457.0 | 1689.1 | 2953.1 | 0.00** |
| Multiple fracture | 5 | 1 | 755.0 | 1617.3 | 1530.3 | 1.00 |
|  |  |  | 800.0 | 1616.3 | 1621.5 | 0.90 |
|  |  |  | 1150.0 | 1610.2 | 2330.9 | 0.26 |
|  |  |  | 1353.0 | 1607.7 | 2742.3 | 0.00** |

*sample weight includes weight of experimental casing **effective 0% saturation of fracture. Cement matrix was maintained saturated at all times. Samples when dry: Simple fracture 1687.3 g; multiple fracture 1604.1 g

Calculation for resistivity data in S1 Table:

The impedance measurements were made using RCON2 (Giatec Scientific Inc., Ottawa, ON) and converted to resistivity by multiplying the geometry of the monolith.

$\rho=Z\cdot\frac{A}{L}$ *eqn S1*

Where: ρ = resistivity; Z = impedance; A = cross sectional area; L = length

A linear relationship was generated with the calibration data and used to indirectly determine the saturation of fractures.
