# Supplementary material for: Relative permeability for water and gas through fractures in cement
Source: PLoS One. 2019 Jan 23;14(1):e0210741. doi: 10.1371/journal.pone.0210741 (PMC6343898; doi:10.1371/journal.pone.0210741)
Supplement: S2 Table — Data measured from relative permeability experiments. (DOCX) [file pone.0210741.s002.docx]

**S2 Table.** Raw data. Data measured from relative permeability experiments.

| **Sample** | **Flow direction** | **P water (Pa)** | **P air (Pa)** | **Volume (cm^3^)** | **time (s)** | **Air flow (cm^3^ s^-1^)** | **Frequency (KHz)** | **voltage (Ω)** | **angle (°)** | **Resistivity** |
| --- | --- | --- | --- | --- | --- | --- | --- | --- | --- | --- |
| simple | horizontal | 120306.8 | n/a | 9.3812 | 32.75 | 0 | 5 | 966 | 0 | 1957.9 |
| simple | horizontal | 120306.8 | n/a | 8.982 | 31.27 | 0 | 5 | 967 | 0 | 1959.9 |
| simple | horizontal | 120306.8 | n/a | 9.2814 | 32.4 | 0 | 5 | 966 | 0 | 1957.9 |
| simple | horizontal | 116309.6 | 112312.4 | 6.2 | 44.2 | 1.0 | 5.0 | 1040.0 | 0.0 | 2107.9 |
| simple | horizontal | 116309.6 | 112312.4 | 4.1 | 29.3 | 1.0 | 5.0 | 1040.0 | 0.0 | 2107.9 |
| simple | horizontal | 116309.6 | 112312.4 | 4.0 | 28.3 | 1.0 | 5.0 | 1040.0 | 0.0 | 2107.9 |
| simple | horizontal | 115809.9 | 112312.4 | 3.6 | 31.7 | 1.7 | 5.0 | 1050.0 | 0.0 | 2128.2 |
| simple | horizontal | 115809.9 | 112312.4 | 3.3 | 28.5 | 1.7 | 5.0 | 1050.0 | 0.0 | 2128.2 |
| simple | horizontal | 115809.9 | 112312.4 | 3.0 | 26.3 | 1.7 | 5.0 | 1050.0 | 0.0 | 2128.2 |
| simple | horizontal | 115310.3 | 112312.4 | 3.5 | 41.1 | 2.2 | 5.0 | 1060.0 | 0.0 | 2148.4 |
| simple | horizontal | 115310.3 | 112312.4 | 2.8 | 33.3 | 2.2 | 5.0 | 1060.0 | 0.0 | 2148.4 |
| simple | horizontal | 115310.3 | 112312.4 | 2.6 | 29.3 | 2.2 | 5.0 | 1060.0 | 0.0 | 2148.4 |
| simple | horizontal | 114810.6 | 112312.4 | 1.1 | 46.3 | 3.7 | 5.0 | 1090.0 | 0.0 | 2209.2 |
| simple | horizontal | 114810.6 | 112312.4 | 1.1 | 48.3 | 3.7 | 5.0 | 1090.0 | 0.0 | 2209.2 |
| simple | horizontal | 114810.6 | 112312.4 | 1.2 | 53.4 | 3.8 | 5.0 | 1090.0 | 0.0 | 2209.2 |
| simple | horizontal | 114510.8 | 112312.4 | 0.5 | 99.3 | 4.5 | 5.0 | 1180.0 | 0.0 | 2391.7 |
| simple | horizontal | 114510.8 | 112312.4 | 0.5 | 86.8 | 4.7 | 5.0 | 1180.0 | 0.0 | 2391.7 |
| simple | horizontal | 114510.8 | 112312.4 | 0.5 | 91.6 | 4.7 | 5.0 | 1180.0 | 0.0 | 2391.7 |
| simple | horizontal | 117308.9 | 112312.4 | 4.7 | 28.9 | 1.0 | 5.0 | 1030.0 | 0.0 | 2087.6 |
| simple | horizontal | 117308.9 | 112312.4 | 4.3 | 26.6 | 1.0 | 5.0 | 1030.0 | 0.0 | 2087.6 |
| simple | horizontal | 117308.9 | 112312.4 | 5.0 | 31.7 | 1.0 | 5.0 | 1030.0 | 0.0 | 2087.6 |
| simple | horizontal | 117308.9 | 112812.0 | 3.8 | 28.6 | 1.5 | 5.0 | 1030.0 | 0.0 | 2087.6 |
| simple | horizontal | 117308.9 | 112812.0 | 3.5 | 27.1 | 1.5 | 5.0 | 1030.0 | 0.0 | 2087.6 |
| simple | horizontal | 117308.9 | 112812.0 | 3.7 | 28.9 | 1.5 | 5.0 | 1030.0 | 0.0 | 2087.6 |
| simple | horizontal | 117308.9 | 113311.7 | 4.3 | 41.3 | 2.2 | 5.0 | 1050.0 | 0.0 | 2128.2 |
| simple | horizontal | 117308.9 | 113311.7 | 3.4 | 32.0 | 2.3 | 5.0 | 1050.0 | 0.0 | 2128.2 |
| simple | horizontal | 117308.9 | 113311.7 | 2.9 | 27.6 | 2.0 | 5.0 | 1050.0 | 0.0 | 2128.2 |
| simple | horizontal | 117308.9 | 113811.3 | 2.6 | 35.4 | 2.8 | 5.0 | 1070.0 | 0.0 | 2168.7 |
| simple | horizontal | 117308.9 | 113811.3 | 2.5 | 33.5 | 3.0 | 5.0 | 1070.0 | 0.0 | 2168.7 |
| simple | horizontal | 117308.9 | 113811.3 | 2.3 | 31.1 | 3.0 | 5.0 | 1070.0 | 0.0 | 2168.7 |
| simple | horizontal | 117308.9 | 114311.0 | 2.2 | 42.7 | 3.7 | 5.0 | 1060.0 | 0.0 | 2148.4 |
| simple | horizontal | 117308.9 | 114311.0 | 2.1 | 38.0 | 3.7 | 5.0 | 1060.0 | 0.0 | 2148.4 |
| simple | horizontal | 117308.9 | 114311.0 | 2.2 | 43.3 | 3.7 | 5.0 | 1060.0 | 0.0 | 2148.4 |
| simple | horizontal | 117308.9 | 114810.6 | 1.6 | 55.5 | 4.7 | 5.0 | 1100.0 | 0.0 | 2229.5 |
| simple | horizontal | 117308.9 | 114810.6 | 1.6 | 63.5 | 4.8 | 5.0 | 1100.0 | 0.0 | 2229.5 |
| simple | horizontal | 117308.9 | 114810.6 | 1.8 | 69.6 | 4.5 | 5.0 | 1100.0 | 0.0 | 2229.5 |
| simple | horizontal | 117308.9 | 115310.3 | 0.7 | 91.0 | 6.5 | 5.0 | 1190.0 | 0.0 | 2411.9 |
| simple | horizontal | 117308.9 | 115310.3 | 0.8 | 99.9 | 6.3 | 5.0 | 1190.0 | 0.0 | 2411.9 |
| simple | horizontal | 117308.9 | 115310.3 | 0.7 | 87.0 | 6.0 | 5.0 | 1190.0 | 0.0 | 2411.9 |
| simple | horizontal | 121306.1 | 114810.6 | 7.9 | 33.5 | 0.8 | 5.0 | 1030.0 | 0.0 | 2087.6 |
| simple | horizontal | 121306.1 | 114810.6 | 7.7 | 32.4 | 0.8 | 5.0 | 1030.0 | 0.0 | 2087.6 |
| simple | horizontal | 121306.1 | 115310.3 | 6.1 | 28.7 | 1.0 | 5.0 | 1030.0 | 0.0 | 2087.6 |
| simple | horizontal | 121306.1 | 115310.3 | 5.7 | 26.9 | 1.2 | 5.0 | 1030.0 | 0.0 | 2087.6 |
| simple | horizontal | 121306.1 | 115310.3 | 5.9 | 27.3 | 1.2 | 5.0 | 1030.0 | 0.0 | 2087.6 |
| simple | horizontal | 121306.1 | 115809.9 | 5.5 | 29.2 | 1.7 | 5.0 | 1040.0 | 0.0 | 2107.9 |
| simple | horizontal | 121306.1 | 115809.9 | 4.9 | 27.2 | 1.7 | 5.0 | 1040.0 | 0.0 | 2107.9 |
| simple | horizontal | 121306.1 | 115809.9 | 5.9 | 31.8 | 1.7 | 5.0 | 1040.0 | 0.0 | 2107.9 |
| simple | horizontal | 121306.1 | 116309.6 | 4.8 | 29.4 | 2.0 | 5.0 | 1050.0 | 0.0 | 2128.2 |
| simple | horizontal | 121306.1 | 116309.6 | 5.4 | 33.9 | 2.0 | 5.0 | 1050.0 | 0.0 | 2128.2 |
| simple | horizontal | 121306.1 | 116309.6 | 4.8 | 29.8 | 2.0 | 5.0 | 1050.0 | 0.0 | 2128.2 |
| simple | horizontal | 121306.1 | 116809.2 | 4.8 | 34.8 | 2.8 | 5.0 | 1060.0 | 0.0 | 2148.4 |
| simple | horizontal | 121306.1 | 116809.2 | 3.7 | 27.2 | 2.8 | 5.0 | 1060.0 | 0.0 | 2148.4 |
| simple | horizontal | 121306.1 | 116809.2 | 3.8 | 28.1 | 2.8 | 5.0 | 1050.0 | 0.0 | 2128.2 |
| simple | horizontal | 121306.1 | 117308.9 | 3.5 | 29.9 | 3.3 | 5.0 | 1060.0 | 0.0 | 2148.4 |
| simple | horizontal | 121306.1 | 117308.9 | 3.3 | 28.8 | 3.3 | 5.0 | 1050.0 | 0.0 | 2128.2 |
| simple | horizontal | 121306.1 | 117308.9 | 3.0 | 26.6 | 3.7 | 5.0 | 1070.0 | 0.0 | 2168.7 |
| simple | vertical | 122805.1 | 116309.6 | 12.0 | 46.0 | 1.5 | 5.0 | 965.0 | 0.0 | 1955.9 |
| simple | vertical | 122805.1 | 116309.6 | 7.5 | 28.9 | 1.7 | 5.0 | 968.0 | 0.0 | 1962.0 |
| simple | vertical | 120306.8 | 116309.6 | 3.6 | 26.6 | 3.0 | 5.0 | 1010.0 | 1.0 | 2047.1 |
| simple | vertical | 120306.8 | 116309.6 | 3.8 | 27.5 | 3.0 | 5.0 | 1020.0 | 1.0 | 2067.4 |
| simple | vertical | 120306.8 | 114311.0 | 6.0 | 27.1 | 1.3 | 5.0 | 1000.0 | 1.0 | 2026.8 |
| simple | vertical | 120306.8 | 114311.0 | 7.0 | 30.9 | 1.3 | 5.0 | 1000.0 | 1.0 | 2026.8 |
| simple | vertical | 131299.2 | 119307.5 | 12.7 | 27.7 | 0.2 | 5.0 | 993.0 | 1.0 | 2012.6 |
| simple | vertical | 131299.2 | 119307.5 | 14.1 | 30.8 | 0.2 | 5.0 | 993.0 | 1.0 | 2012.6 |
| simple | vertical | 126302.7 | 119307.5 | 9.8 | 35.6 | 2.8 | 5.0 | 1020.0 | 1.0 | 2067.4 |
| simple | vertical | 126302.7 | 119307.5 | 7.8 | 28.1 | 3.0 | 5.0 | 1020.0 | 1.0 | 2067.4 |
| simple | vertical | 125303.3 | 119307.5 | 5.9 | 26.0 | 4.0 | 5.0 | 1030.0 | 1.0 | 2087.6 |
| simple | vertical | 124304.0 | 119307.5 | 5.2 | 28.3 | 4.5 | 5.0 | 1020.0 | 1.0 | 2067.4 |
| simple | vertical | 123304.7 | 119307.5 | 3.8 | 28.3 | 5.7 | 5.0 | 1030.0 | 1.0 | 2087.6 |
| simple | vertical | 122305.4 | 119307.5 | 3.2 | 38.4 | 6.7 | 5.0 | 1050.0 | 1.0 | 2128.2 |
| simple | vertical | 121306.1 | 119307.5 | 1.6 | 43.0 | 8.8 | 5.0 | 1070.0 | 1.0 | 2168.7 |
| simple | vertical | 121306.1 | 119307.5 | 1.7 | 43.6 | 8.7 | 5.0 | 1070.0 | 1.0 | 2168.7 |
| simple | vertical | 121306.1 | 119307.5 | 1.7 | 42.5 | 8.7 | 5.0 | 1070.0 | 1.0 | 2168.7 |
| simple | vertical | 120806.5 | 118308.2 | 0.8 | 66.6 | 15.0 | 5.0 | 1230.0 | 0.0 | 2493.0 |
| simple | vertical | 120806.5 | 118308.2 | 0.8 | 66.6 | 15.0 | 5.0 | 1270.0 | 0.0 | 2574.1 |
| simple | vertical | 121306.1 | 116309.6 | 6.9 | 31.2 | 1.5 | 5.0 | 1030.0 | 0.0 | 2087.6 |
| simple | vertical | 121306.1 | 116309.6 | 6.7 | 30.5 | 1.3 | 5.0 | 1030.0 | 0.0 | 2087.6 |
| simple | vertical | 121306.1 | 117308.9 | 4.9 | 28.2 | 2.3 | 5.0 | 1040.0 | 0.0 | 2107.9 |
| simple | vertical | 121306.1 | 117308.9 | 5.0 | 28.6 | 2.3 | 5.0 | 1040.0 | 0.0 | 2107.9 |
| simple | vertical | 121306.1 | 118308.2 | 3.4 | 27.8 | 3.2 | 5.0 | 1040.0 | 0.0 | 2107.9 |
| simple | vertical | 121306.1 | 118308.2 | 4.0 | 31.7 | 3.3 | 5.0 | 1040.0 | 0.0 | 2107.9 |
| simple | vertical | 121306.1 | 119307.5 | 2.4 | 31.6 | 10.0 | 5.0 | 1060.0 | 0.0 | 2148.4 |
| simple | vertical | 121306.1 | 119307.5 | 2.5 | 33.0 | 10.5 | 5.0 | 1050.0 | 0.0 | 2128.2 |
| simple | vertical | 121306.1 | 119807.2 | 1.7 | 27.9 | 13.5 | 5.0 | 1060.0 | 0.0 | 2148.4 |
| simple | vertical | 121306.1 | 118308.2 | 2.0 | 32.7 | 15.0 | 5.0 | 1050.0 | 0.0 | 2128.2 |
| simple | vertical | 121306.1 | 120306.8 | 1.9 | 52.8 | 15.5 | 5.0 | 1080.0 | 0.0 | 2189.0 |
| simple | vertical | 121306.1 | 120306.8 | 2.1 | 52.4 | 15.2 | 5.0 | 1080.0 | 0.0 | 2189.0 |
| simple | vertical | 121306.1 | 120806.5 | 1.5 | 68.0 | 15.8 | 5.0 | 1110.0 | 0.0 | 2249.8 |
| simple | vertical | 121306.1 | 120806.5 | 1.7 | 81.9 | 15.5 | 5.0 | 1100.0 | 0.0 | 2229.5 |
| simple | vertical | 123304.7 | 121306.1 | 4.0 | 47.8 | 8.7 | 5.0 | 1060.0 | 0.0 | 2148.4 |
| simple | vertical | 123304.7 | 121306.1 | 2.7 | 29.8 | 8.7 | 5.0 | 1060.0 | 0.0 | 2148.4 |
| simple | vertical | 123304.7 | 122305.4 | 1.5 | 30.7 | 11.5 | 5.0 | 1080.0 | 0.0 | 2189.0 |
| simple | vertical | 123304.7 | 122305.4 | 1.7 | 32.8 | 11.7 | 5.0 | 1080.0 | 0.0 | 2189.0 |
| simple | vertical | 111313.1 | 105713.9 | 11.0 | 48.0 | 0.0 | 5.0 | 762.0 | 0.0 | 1544.4 |
| simple | vertical | 111313.1 | 105713.9 | 25.0 | 110.9 | 0.0 | 5.0 | 762.0 | 0.0 | 1544.4 |
| simple | vertical | 111313.1 | 105713.9 | 11.0 | 47.0 | 0.0 | 5.0 | 762.0 | 0.0 | 1544.4 |
| simple | vertical | 111313.1 | 105713.9 | 25.0 | 110.8 | 0.0 | 5.0 | 763.0 | 0.0 | 1546.5 |
| simple | vertical | 109314.4 | 105314.5 | 9.0 | 68.0 | 0.0 | 5.0 | 780.0 | 0.0 | 1580.9 |
| simple | vertical | 109314.4 | 105314.5 | 13.0 | 103.0 | 0.0 | 5.0 | 780.0 | 0.0 | 1580.9 |
| simple | vertical | 109314.4 | 105314.5 | 17.0 | 134.0 | 0.0 | 5.0 | 780.0 | 0.0 | 1580.9 |
| simple | vertical | 109314.4 | 105314.5 | 25.0 | 206.6 | 0.0 | 5.0 | 780.0 | 0.0 | 1580.9 |
| simple | vertical | 108315.1 | 105314.5 | 5.0 | 96.0 | 0.0 | 5.0 | 821.0 | 0.0 | 1664.0 |
| simple | vertical | 108315.1 | 105314.5 | 7.0 | 136.0 | 0.0 | 5.0 | 821.0 | 0.0 | 1664.0 |
| simple | vertical | 108315.1 | 105314.5 | 9.0 | 181.0 | 0.0 | 5.0 | 821.0 | 0.0 | 1664.0 |
| simple | vertical | 108315.1 | 105314.5 | 11.0 | 225.2 | 0.0 | 5.0 | 821.0 | 0.0 | 1664.0 |
| simple | vertical | 109314.4 | 105314.5 | 5.0 | 40.2 | 0.0 | 5.0 | 785.0 | 0.0 | 1591.1 |
| simple | vertical | 111313.1 | 106812.4 | 7.0 | 47.3 | 0.0 | 5.0 | 784.0 | 0.0 | 1589.0 |
| simple | vertical | 111313.1 | 107311.7 | 5.0 | 40.9 | 0.0 | 5.0 | 787.0 | 0.0 | 1595.1 |
| simple | vertical | 108315.1 | 104815.2 | 5.0 | 68.8 | 0.0 | 5.0 | 799.0 | 0.0 | 1619.4 |
| simple | vertical | 107315.8 | 104315.9 | 3.0 | 70.1 | 0.0 | 5.0 | 813.0 | 0.0 | 1647.8 |
| simple | vertical | 111313.1 | 107811.0 | 11.0 | 58.0 | 0.0 | 5.0 | 762.0 | 0.0 | 1544.4 |
| simple | vertical | 111313.1 | 107811.0 | 19.0 | 102.9 | 0.0 | 5.0 | 762.0 | 0.0 | 1544.4 |
| simple | vertical | 111313.1 | 107411.6 | 7.0 | 53.0 | 0.0 | 5.0 | 789.0 | 0.0 | 1599.2 |
| simple | vertical | 111313.1 | 107411.6 | 11.0 | 85.3 | 0.0 | 5.0 | 789.0 | 0.0 | 1599.2 |
| simple | vertical | 109814.1 | 106113.4 | 7.0 | 65.7 | 0.0 | 5.0 | 798.0 | 0.0 | 1617.4 |
| simple | vertical | 108814.8 | 105614.1 | 5.0 | 68.0 | 0.0 | 5.0 | 810.0 | 0.0 | 1641.7 |
| simple | vertical | 107815.5 | 104815.2 | 5.0 | 102.5 | 0.0 | 5.0 | 823.0 | 0.0 | 1668.1 |
| simple | vertical | 106816.2 | 104216.0 | 3.0 | 141.7 | 0.0 | 5.0 | 846.0 | 0.0 | 1714.7 |
| simple | vertical | 106316.5 | 104016.3 | 1.0 | 330.3 | 0.0 | 5.0 | 900.0 | 0.0 | 1824.1 |
| multiple | horizontal | 111313.1 | n/a | 8.3 | 22.4 | 0.0 | 5.0 | 755.0 | 0.0 | 1530.3 |
| multiple | horizontal | 111313.1 | n/a | 12.9 | 35.1 | 0.0 | 5.0 | 764.0 | 0.0 | 1548.5 |
| multiple | horizontal | 121306.1 | n/a | 22.0 | 33.5 | 0.0 | 5.0 | 755.0 | 0.0 | 1530.3 |
| multiple | horizontal | 121306.1 | n/a | 22.9 | 35.2 | 0.0 | 5.0 | 758.0 | 0.0 | 1536.3 |
| multiple | horizontal | 131299.2 | n/a | 19.4 | 22.4 | 0.0 | 5.0 | 757.0 | 0.0 | 1534.3 |
| multiple | horizontal | 131299.2 | n/a | 25.0 | 29.0 | 0.0 | 5.0 | 756.0 | 0.0 | 1532.3 |
| multiple | horizontal | 141292.2 | n/a | 31.7 | 30.9 | 0.0 | 5.0 | 747.0 | 0.0 | 1514.0 |
| multiple | horizontal | 141292.2 | n/a | 25.9 | 25.0 | 0.0 | 5.0 | 748.0 | 0.0 | 1516.1 |
| multiple | horizontal | 101320.0 | 121306.1 | 0.0 | 0.0 | 14.5 | 5.0 | 1410.0 | 1.0 | 2857.8 |
| multiple | horizontal | 113311.7 | 101320.0 | 17.3 | 35.4 | 0.0 | 5.0 | 730.0 | 0.0 | 1479.6 |
| multiple | horizontal | 113311.7 | 101320.0 | 12.2 | 27.1 | 0.0 | 5.0 | 732.0 | 0.0 | 1483.6 |
| multiple | horizontal | 101320.0 | 118308.2 | 0.0 | 0.0 | 23.8 | 5.0 | 1280.0 | 1.0 | 2594.3 |
| multiple | horizontal | 101320.0 | 118308.2 | 0.0 | 0.0 | 24.8 | 5.0 | 1370.0 | 1.0 | 2776.8 |
| multiple | horizontal | 111313.1 | 105317.2 | 6.9 | 28.6 | 1.0 | 5.0 | 865.0 | 0.0 | 1753.2 |
| multiple | horizontal | 111313.1 | 105317.2 | 8.7 | 36.4 | 1.0 | 5.0 | 865.0 | 0.0 | 1753.2 |
| multiple | horizontal | 121306.1 | 108814.8 | 14.6 | 29.1 | 2.3 | 5.0 | 855.0 | 0.0 | 1732.9 |
| multiple | horizontal | 121306.1 | 108814.8 | 14.1 | 28.5 | 2.3 | 5.0 | 856.0 | 0.0 | 1735.0 |
| multiple | horizontal | 123304.7 | 108814.8 | 15.9 | 28.8 | 2.2 | 5.0 | 845.0 | 0.0 | 1712.7 |
| multiple | horizontal | 123304.7 | 108814.8 | 15.1 | 27.3 | 2.2 | 5.0 | 845.0 | 0.0 | 1712.7 |
| multiple | horizontal | 125303.3 | 108814.8 | 18.4 | 30.4 | 2.3 | 5.0 | 832.0 | 0.0 | 1686.3 |
| multiple | horizontal | 125303.3 | 108814.8 | 22.6 | 37.7 | 2.3 | 5.0 | 832.0 | 0.0 | 1686.3 |
| multiple | horizontal | 125303.3 | 111313.1 | 14.6 | 27.6 | 2.8 | 5.0 | 855.0 | 0.0 | 1732.9 |
| multiple | horizontal | 125303.3 | 111313.1 | 14.6 | 27.5 | 3.0 | 5.0 | 858.0 | 0.0 | 1739.0 |
| multiple | horizontal | 125303.3 | 112312.4 | 14.5 | 28.2 | 3.7 | 5.0 | 860.0 | 0.0 | 1743.1 |
| multiple | horizontal | 125303.3 | 112312.4 | 14.0 | 27.3 | 3.7 | 5.0 | 860.0 | 0.0 | 1743.1 |
| multiple | horizontal | 125303.3 | 113311.7 | 16.0 | 33.3 | 4.7 | 5.0 | 867.0 | 0.0 | 1757.3 |
| multiple | horizontal | 125303.3 | 113311.7 | 18.9 | 39.2 | 4.7 | 5.0 | 874.0 | 0.0 | 1771.4 |
| multiple | horizontal | 125303.3 | 113311.7 | 15.3 | 31.5 | 4.7 | 5.0 | 872.0 | 0.0 | 1767.4 |
| multiple | horizontal | 125303.3 | 114311.0 | 13.7 | 30.2 | 3.3 | 5.0 | 877.0 | 0.0 | 1777.5 |
| multiple | horizontal | 125303.3 | 114311.0 | 22.3 | 48.7 | 3.8 | 5.0 | 875.0 | 0.0 | 1773.5 |
| multiple | horizontal | 125303.3 | 115310.3 | 12.0 | 27.4 | 4.7 | 5.0 | 879.0 | 0.0 | 1781.6 |
| multiple | horizontal | 125303.3 | 115310.3 | 13.9 | 31.1 | 4.7 | 5.0 | 877.0 | 0.0 | 1777.5 |
| multiple | horizontal | 125303.3 | 116309.6 | 16.6 | 39.1 | 4.7 | 5.0 | 881.0 | 0.0 | 1785.6 |
| multiple | horizontal | 121306.1 | 114311.0 | 14.5 | 41.8 | 7.5 | 5.0 | 863.0 | 1.0 | 1749.2 |
| multiple | horizontal | 121306.1 | 114311.0 | 10.9 | 31.2 | 7.5 | 5.0 | 864.0 | 1.0 | 1751.2 |
| multiple | horizontal | 120306.8 | 114311.0 | 9.0 | 28.6 | 8.0 | 5.0 | 873.0 | 0.0 | 1769.4 |
| multiple | horizontal | 120306.8 | 114311.0 | 14.2 | 46.0 | 8.0 | 5.0 | 878.0 | 0.0 | 1779.6 |
| multiple | horizontal | 119307.5 | 114311.0 | 8.5 | 31.2 | 10.0 | 5.0 | 889.0 | 0.0 | 1801.9 |
| multiple | horizontal | 119307.5 | 114311.0 | 6.9 | 25.4 | 10.0 | 5.0 | 890.0 | 0.0 | 1803.9 |
| multiple | horizontal | 117308.9 | 112312.4 | 7.1 | 27.6 | 8.3 | 5.0 | 888.0 | 1.0 | 1799.8 |
| multiple | horizontal | 117308.9 | 112312.4 | 7.8 | 30.4 | 8.3 | 5.0 | 890.0 | 1.0 | 1803.9 |
| multiple | horizontal | 115310.3 | 111313.1 | 6.2 | 29.8 | 8.3 | 5.0 | 905.0 | 0.0 | 1834.3 |
| multiple | horizontal | 115310.3 | 111313.1 | 6.1 | 29.0 | 8.7 | 5.0 | 905.0 | 1.0 | 1834.3 |
| multiple | horizontal | 114311.0 | 111313.1 | 4.8 | 28.3 | 10.0 | 5.0 | 925.0 | 1.0 | 1874.8 |
| multiple | horizontal | 114311.0 | 111313.1 | 4.7 | 28.4 | 10.0 | 5.0 | 927.0 | 1.0 | 1878.9 |
| multiple | horizontal | 113311.7 | 112312.4 | 3.6 | 27.8 | 13.3 | 5.0 | 941.0 | 1.0 | 1907.2 |
| multiple | horizontal | 113311.7 | 112312.4 | 3.9 | 31.0 | 13.7 | 5.0 | 940.0 | 1.0 | 1905.2 |
| multiple | horizontal | 112312.4 | 112812.0 | 2.6 | 30.1 | 15.8 | 5.0 | 999.0 | 1.0 | 2024.8 |
| multiple | horizontal | 111313.1 | 112812.0 | 1.0 | 59.5 | 15.8 | 5.0 | 1070.0 | 0.0 | 2168.7 |
| multiple | horizontal | 111313.1 | 112812.0 | 1.1 | 66.5 | 16.0 | 5.0 | 1090.0 | 0.0 | 2209.2 |
| multiple | horizontal | 110313.8 | 112312.4 | 1.0 | 105.2 | 16.7 | 5.0 | 1130.0 | 1.0 | 2290.3 |
| multiple | horizontal | 109314.4 | 110813.4 | 0.6 | 191.4 | 16.7 | 5.0 | 1200.0 | 1.0 | 2432.2 |
| multiple | horizontal | 109314.4 | 110813.4 | 0.6 | 189.3 | 16.7 | 5.0 | 1220.0 | 1.0 | 2472.7 |
