# Supplementary material for: Relative permeability for water and gas through fractures in cement
Source: PLoS One. 2019 Jan 23;14(1):e0210741. doi: 10.1371/journal.pone.0210741 (PMC6343898; doi:10.1371/journal.pone.0210741)
Supplement: S1 Fig — Blue triangles are simple fracture and black circles are multiple fracture sample. (DOCX) [file pone.0210741.s003.docx]

**S1 Fig**. Regression of resistivity saturation calibration from Table S1. Blue triangles are simple fracture and black circles are multiple fracture sample.
