# Supplementary material for: Relative permeability for water and gas through fractures in cement
Source: PLoS One. 2019 Jan 23;14(1):e0210741. doi: 10.1371/journal.pone.0210741 (PMC6343898; doi:10.1371/journal.pone.0210741)
Supplement: S2 Fig — Monolith was wrapped in polyolefin tubing with stainless steel end caps (with influent and effluent ports) attached. Air pumps supplied air to precision flow controllers, where one supplied air directly to the monolith influent end cap and the other to a sealed reservoir. Head pressure to the influent end was controlled by maintaining the reservoir at a constant gauge pressure (10–40 kPa). Resistivity meter electrodes were attached to the steel pressure fittings on the end caps. Effluent eluted from one port into a beaker, which acted as a phase separator, on a scale. Beaker was sealed with a tube to an air flow monitor. (DOCX) [file pone.0210741.s004.docx]

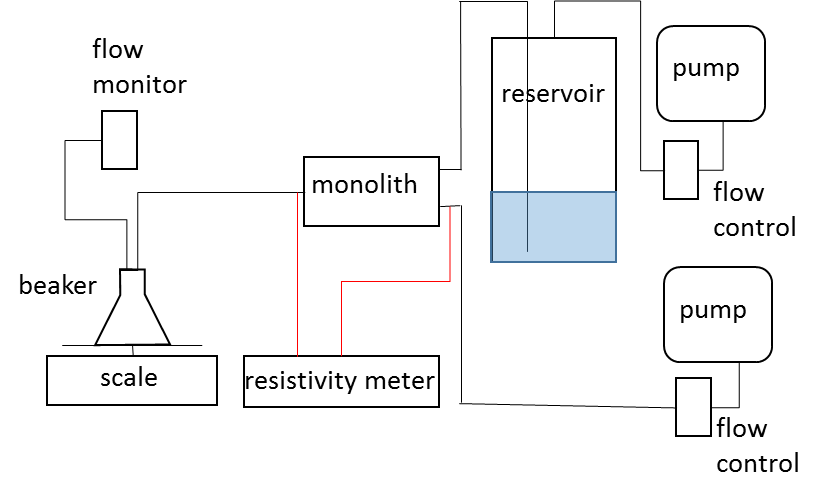


**S2 Fig**. Schematic of experimental design. Monolith (cylinder) was wrapped in polyolefin tubing with stainless steel end caps (with influent and effluent ports) attached. Air pumps supplied air to precision flow controllers, where one supplied air directly to the monolith influent end cap and the other to a sealed reservoir. Head pressure to the influent end was controlled by maintaining the reservoir at a constant gauge pressure (10-40 kPa). Resistivity meter electrodes were attached to the steel pressure fittings on the end caps. Effluent eluted from one port into a beaker, which acted as a phase separator, on a scale. Beaker was sealed with a tube to an air flow monitor.
