# Supplementary material for: Relative permeability for water and gas through fractures in cement
Source: PLoS One. 2019 Jan 23;14(1):e0210741. doi: 10.1371/journal.pone.0210741 (PMC6343898; doi:10.1371/journal.pone.0210741)
Supplement: S3 Fig — (DOCX) [file pone.0210741.s005.docx]

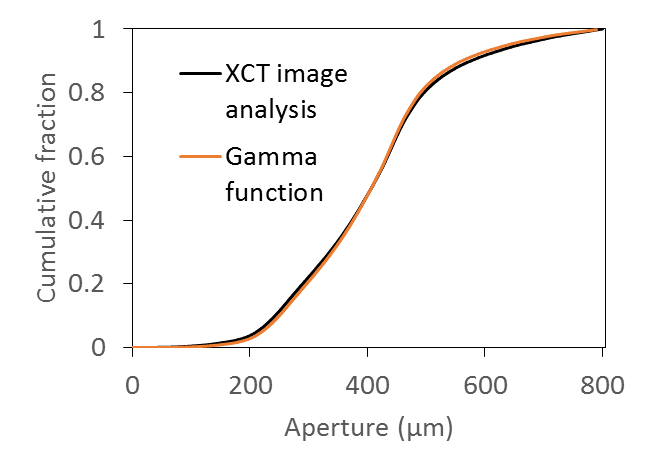


**S3 Fig.** Cumulative fraction of aperture size from computational segmented fracture and calculated gamma function, Eqn 14.
